# Sleep quality and mental disorder symptoms among correctional workers in Ontario, Canada

**DOI:** 10.1038/s41598-024-65891-8

**Published:** 2024-06-28

**Authors:** Rosemary Ricciardelli, Tamara L. Taillieu, Megan McElheran, Heidi Cramm, Harsha Ajith, Matthew S. Johnston, R. Nicholas Carleton

**Affiliations:** 1 School of Maritime Studies, Fisheries and Marine Institute, Memorial University of Newfoundland, 155 Ridge Road, St. John’s, NL A1C 5R3 Canada; 2 Department of Community Health Sciences, University of Manitoba, Winnipeg, Canada; 3 Wayfound Mental Health Group, Regina, Canada; 4 School of Rehabilitation Therapy, Queen’s University, Kingston, Canada; 5 Memorial University of Newfoundland, St. John’s, Canada; 6 School of Maritime Studies, Fisheries and Marine Institute, Memorial University of Newfoundland, St. John’s, Canada; 7 Department of Psychology, University of Regina, Regina, Canada

**Keywords:** Sleep, Correctional workers, Canada, Mental health and well-being, Physiology, Psychology

## Abstract

Correctional workers (CWs) report high levels of work stressors, frequent exposures to potentially psychologically traumatic events (PPTEs), and substantial mental health challenges. There is evidence of associations between sleep disturbances and diverse mental health challenges, including preliminary evidence from public safety personnel; however, replications and extensions would better inform interventions to support mental health. The current study was designed to examine associations between quality of sleep, work stress, and mental health disorders in a sample of diverse CWs employed in a provincial correctional service in Ontario, Canada. Data were analyzed from 943 CWs who participated in the cross-sectional, web-based Ontario Provincial Correctional Worker Mental Health and Well-Being Study conducted from December 2017 to June 2018. Sleep quality indicators included symptoms of insomnia, total hours of sleep per night on work nights and off-shift nights, number of days feeling rested per week, and overall sleep quality. Descriptive statistics, analyses of variance, correlational analyses, and logistic regression were used to examine relationships among sleep quality, stress of shift work, and mental health disorder symptoms. CWs slept an average of 6.0 h per night when working and 7.2 h during off-shift nights. CWs reported waking up feeling rested an average of 2.6 days per week and rated their overall quality of sleep in the fair to poor range. Many CWs (64.9%) screened positive for clinically significant symptoms of insomnia. There were also differences across occupational groups such that CWs working as correctional officers reported the most sleep problems. There were statistically significant relationships between insomnia and mental health disorder symptoms. Higher levels of stress from shift work were associated with worse sleep quality. CWs, especially those working as correctional officers in a provincial prison, reported many indicators consistent with poorer quality of sleep. Poor quality of sleep was also associated with work stress and mental health disorders.

## Introduction

Correctional workers (CWs) serving in community or institutional settings are public safety personnel (PSP) who must navigate an often stressful and largely unpredictable workplace^1–5^. In the current article, CWs refer to anyone working in community (e.g., probation and parole officers), institutional (e.g., correctional and program officers), or administrative (e.g., support to wardens, payroll) correctional services in the Canadian province of Ontario. Across CW positions, exposures to potentially psychologically traumatic events (PPTEs) are commonplace^5–10^. PPTE exposures can have highly variable impacts, with previous evidence indicating that sudden violent or accidental death, physical assault, harassment, and fire or explosions are particularly compromising for CWs^6,10^. Our data were collected prior to the COVID-19 pandemic; nevertheless, the COVID-19 pandemic appeared to have further compromised the physical health and safety of CWs^11^, potentially amplifying the extant mental health challenges^11^, though other evidence indicates the prevalence of mental health disorders among CWs was similar during pre- and post-COVID-19 periods^12^.

There are relationships between PPTE and the prevalence of mental health disorders^6,10^. Evidence from a large sample of CWs employed in Ontario provincial correctional services indicated more than half (56.9%) screened positive for one or more mental health disorder(s), with higher rates for women than for men^13^. Participating CWs screened positive for Posttraumatic Stress Disorder (PTSD; 30.7%), Major Depressive Disorder (MDD; 37%), and Generalized Anxiety Disorder (GAD; 30.5%)^13^. There were differences in positive screening proportions among the CW professional categories for correctional officers (59.0%), probation and parole officers (63.2%), institutional governance employees (61.0%), institutional wellness staff (43.7%), institutional training staff (50.0%), and institutional administrative staff (52.0%)^13^.

Increasingly robust evidence suggests CWs report an extremely high prevalence of exposure to PPTEs^6,10^, including PPTEs that are specific to correctional work^14^. These exposures appear to negatively impact CW mental health^6,10^ and preliminary results suggest important interactions with sleep^15^. We designed the current study to better understand the relationships between sleep as interacting with mental health among CWs across different occupational categories.

### Sleep and correctional work

Mental health conditions negatively impact quality and quantity of sleep^16^, as do certain behavioural health and lifestyle habits (e.g., poor stress management, inconsistent eating patterns). A recent PSP study including CWs evidenced 55% of PSP report symptoms consistent with a diagnosis of insomnia, and PSP also reported sleeping fewer hours on an average working night compared to the general population^15^. CWs with sleep disturbances reported high prevalence of mental health symptoms^15^, though participants were not stratified by their distinct occupational category. PSP who screened positive for insomnia also had up to 6.95 times the odds of screening positive for PTSD, MDD, GAD, Social Anxiety Disorder, Panic Disorder, as well as alcohol use disorder^15^. A sleep study with the Washington State Department of Corrections employees evidenced that 28 percent of the sample reported sleep apnea, 45 percent reported clinically significant insomnia, and more than half reported sleeping less than two hours between shifts and feeling fatigued constantly^17^. There was also evidence of correlations between sleep problems and PPTE exposures^17^.

In the general population, shift workers who presented with clinical symptoms of insomnia also reported symptoms of anxiety, depression, and fatigue which adversely impacted work productivity and performance^18^. Among PSP, shift work appears inversely associated with the duration and quality of sleep^15^. CW vocational requirements like atypical work times, working outside traditional hours, and very long shifts with overtime all negatively impact sleep quality^4,19^. A study on shift work and its effects on the mental health of CWs documented the complex and interdependent relationships that exist between unpredictable working times, depression symptoms, and the amount of sleep obtained^4^. For example, having little control over work schedules and experiencing increasingly unpredictable work hours were associated with greater symptoms of MDD and mitigated by having sufficient sleep^4^.

Overall, sleep disturbances are associated with a range of mental health challenges and are a diagnostic criteria for several disorders^20^. Sleep disruptions also appear to be risk factors for suicidal ideation, plans, and behaviours^21,22^. The limited extant literature on rotating shift work and mental health challenges among CWs supports important interrelationships^15^; however, the extant literature would benefit from assessments of occupational stress due to shift work and the mental health of CWs across different occupational categories.

### Current study

The current study was designed to examine the relationship between sleep quality and positive screens for mental health disorders in a cross-sectional sample of diverse CWs employed in the provincial correctional services of Ontario, Canada. We expected specific interactions with shift work because CWs working in community or institutional settings may have 24-h responsibilities while on duty, though shift lengths for correctional officers in particular generally range from 8 to 12 h. Nurses generally work 8, 10, or 12 h shifts but may work shifts up to 24 h, based on institutional need. Social workers and addiction counsellors tend to work 10- and 12-h rotating shifts. A better understanding of how sleep and shift work are associated with mental health across diverse CW occupational categories may help to inform meaningfully tailored mental health solutions. Accordingly, we expected that: (1) CWs would report sleep disturbances and lower quality of sleep; and (2) CWs who screened positive for a clinical diagnosis of insomnia would also report more mental health challenges, including positive screens for mental health disorders.

## Method

### Data and sample

Data for the current study were derived from the Ontario Provincial Correctional Worker Mental Health and Well-being Study^10,23^. We collected cross-sectional data through an online survey distributed to Ontario CWs from December 2017 to June 2018. We invited CWs to participate through email invitations sent to all individuals under the employ of Ontario’s Ministry of the Solicitor General working in correctional services (approximately 8000 people) by two agency representatives. The representations were associated with (1) Ontario’s Ministry of the Solicitor General, and (2) the Ontario Public Services Employees Union. Email invitations informed potential participants of the purpose of the survey and provided an anonymous link to each email recipient that routed the employee to the start of the survey, where further information related to informed consent procedures, confidentiality, data storage, risks and benefits, and withdrawal of consent procedures was made available. Because the emails informing potential participants could be forwarded (and the listservs used had an unknown level of overlap), the total number of correctional employees invited to participate or the study response rate cannot be computed, which is a limitation of our study. Further details about the study procedures have been published elsewhere^13,23–25^.

A total of 1487 CWs participated in the data collection. The current study included data from participants who completed the sections on sleep quality and mental health disorders (i.e., data required for the current analysis) and could be definitively placed into one of six occupational groups of interest (*n* = 943): (1) institutional wellness (e.g., nurses, social workers, counsellors); (2) institutional training (e.g., teachers, program officers, chaplains, volunteer coordinators); (3) institutional governance (e.g., superintendents or deputy superintendents); (4) institutional correctional officers; (5) probation and parole officers; and (6) institutional administration (e.g., administrative assistants, payroll and other support roles).

### Patient and public involvement statement

There was no patient involvement, and public involvement was restricted to CWs in the Ontario provincial prison system.

### Measures

#### Sleep quality

The Insomnia Severity Index (ISI) is a 7-item self-report measure designed to assess current (i.e., in the last month) sleep quality^26^. Each item was rated on a 5-point Likert Scale ranging from 0 (very satisfied) to 4 (very dissatisfied). The ISI has good internal consistency, discriminatory capacity, and convergent validity in both community and clinical samples^26,27^. On the ISI, a score of 10 or more has been used to indicate clinically significant sleep distress^26^. The current study also included four single items designed to assess sleep patterns and quality including: (1) total hours of sleep on work nights (i.e., on average, approximately how many hours per night do you sleep, excluding time spent in bed not sleeping, on weeknights, or on-shift periods if you work shift work?); (2) total number of hours of sleep on off-shift nights (i.e., on average, approximately how many hours per night do you sleep, excluding time spent in bed not sleeping, on weekends, or off-shift periods if you work shift work?); (3) number of days per week you feel rested (i.e., on average, how many days per 7 day week do you wake up feeling rested?); and (4) an overall assessment of the quality of sleep (i.e., how would you rate your quality of sleep overall?) rated on a 5-point Likert Scale ranging from 0 (very poor) to 4 (very good).

#### Mental health disorders

Several well-validated self-report screening measures were used to assess different types of mental health disorder symptoms. PTSD symptoms in the past month were assessed with the 20-item PTSD Checklist for DSM-5 (PCL-5)^28^. A positive screen for PTSD was indicated if the participant reported exposure to at least one traumatic event on the Life Events Checklist for DSM-5^28,29^, met minimum criteria on each symptom cluster of the PCL-5, and had a total score > 32 on the PCL-5^28^. MDD symptoms in the past 14 days were assessed with the 9-item Patient Health Questionnaire (PHQ-9)^30^. A positive screen for MDD was indicated by a total score > 9 on the PHQ-9^31^. GAD symptoms in the past 14 days were assessed with the 7-item GAD Scale (GAD-7)^32^. A positive screen was indicated by a total score > 9 on the GAD-7^33^. Panic disorder symptoms in the past 7 days were assessed with the 7-item Panic Disorder Severity Scale (PDSS)^34^. A positive screen for panic disorder was indicated by a total score > 9 on the PDSS^34^. Alcohol use disorder symptoms in the past 12 months were assessed with the 10-item Alcohol Use Disorders Identification Test (AUDIT)^35^. A positive screen for alcohol use disorder was indicated by a total score > 15 on the AUDIT^36^. Participants were also asked to self-report whether they had ever been diagnosed with several other mental health disorders, including persistent depressive disorder, bipolar I, bipolar II, cyclothymic disorder, social anxiety disorder, and obsessive compulsive disorder. A dichotomous variable was created to indicate a positive screen for, or self-report of, any mental health disorder.

### Occupational stress

Occupational stress due to shift work was assessed with a single item (i.e., “Please indicate how much stress shift work has caused you over the past 6 months”) on a 7-point Likert scale ranging from 0 (no stress) to 6 (a lot of stress) from the Operational Police Stress Questionnaire (PSQ-Op)^37^.

### Sociodemographic covariates

Sociodemographic covariates included: sex (i.e., male or female), age grouping (i.e., 20–29 years, 30–39 years, 40–49 years, 50–59 years, 60 years and older), marital status (i.e., married/common law, single, separated/divorced/widowed, remarried), highest level of education (i.e., high school or less, some post-secondary [less than 4 year college/university program], university degree of 4 year college program or higher), urban versus rural work location, total years of service (i.e., less than 4 years, 4–9 years, 10–15 years, more than 15 years), and occupational category (i.e., institutional wellness, institutional training, institutional governance, institutional correctional officers, probation and parole officers, institutional administration).

### Statistical analyses

First, we computed descriptive statistics to examine the distribution of sociodemographic covariates, sleep quality indicators, and mental health indicators in the sample. Second, we tested differences in mean scores associated with the total ISI score and other quality of sleep indicators across occupational categories using ANOVA with Bonferroni post-hoc tests. We also tested for differences in the prevalence of screening positive for insomnia (i.e., score of 10 or more on ISI) across occupational categories by changing the reference groups in a series of logistic regression models. Third, a series of logistic regressions models were run to examine: (1) the relationship between a positive screen for insomnia (dichotomous coding based on cut-off score of 10 or more) on the ISI and any positive mental health disorder screen and (2) the relationship between the total insomnia score on the ISI (total scores can range from 0 to 28) and any positive mental health disorder screen. Logistic regression models were adjusted for level of stress due to shift work scores. Logistic regression models were run in the total sample, and then separately for each occupational category. Fourth, we used correlational analyses to examine the correlation between the total insomnia score on the ISI and total mental health disorder screening measure symptom scores. Correlational analyses were also used to examine the correlation between the other four sleep quality indicators and level of stress due to shift work scores. Complete case analysis was used for all statistical models and Stata (version 16.1) software was used for all analyses. Results were considered statistically significant at *p* < 0.05.

### Ethics approval statement

Ethical approval for the study was obtained from the Queen’s University and Affiliated Health Sciences Centre Research Ethics Board (file #6024787), as well as the Research Ethics Boards at the University of Regina (file #2017-098) and Memorial University of Newfoundland (file #20201330-EX). All methods were performed in accordance with the relevant guidelines and regulations.

### Informed consent

Informed consent was voluntarily obtained from all participants.

## Results

Sociodemographic characteristics of the sample are provided in Table 1. The sample was evenly distributed based on sex (49.2% male, 50.9% female). Most participants were between 30 and 59 years of age (79.3%), living in a married/common-law union (62.8%), had at least some post-secondary education (95.0%), and worked in an urban location (97.5%). Years of service were nearly bimodal (i.e., 29.2% reported less than 4 years; 39.1% reported more than 15 years). Most participants worked as institutional correctional officers (59.3%) or probation/parole officers (16.5%). Fewer participants reported working in the other occupational categories (e.g., 3.1% working in institutional administration; 9.5% working in institutional governance).

**Table 1:** Sociodemographic characteristics among correctional workers in Ontario, Canada.

Sociodemographic characteristics	% (n)
Sex	
Male	49.2 (462)
Female	50.9 (478)
Age	
20–29 years	18.4 (172)
30–39 years	28.9 (270)
40–49 years	26.5 (248)
50–59 years	23.9 (224)
60 years and older	2.4 (22)
Marital status	
Married/common-law	62.8 (583)
Single	19.2 (178)
Separated/divorced/widowed	14.4 (134)
Re-married	3.7 (34)
Education	
High school or less	5.0 (46)
Some post-secondary (less than 4 year college/university program)	47.6 (436)
University degree/4 year college or higher	47.4 (435)
Urban/rural work location	
Urban	97.5 (916)
Rural	2.6 (24)
Years of service	
Less than 4 years	29.2 (272)
4–9 years	12.7 (118)
10–15 years	19.1 (178)
More than 15 years	39.1 (365)
Occupational category	
Institutional wellness	8.1 (76)
Institutional training, chaplains, coordination of volunteer	3.5 (33)
Institutional governance	9.5 (90)
Institutional correctional officers	59.3 (559)
Probation/parole officers	16.5 (156)
Institutional administration	3.1 (29)

Descriptive information regarding sleep quality indicators across occupational categories are provided in Table 2. CWs reported sleeping an average of 6.0 h on work nights and 7.2 h on off-shift nights. There were statistically significant differences in hours slept across occupational groups; specifically, participants working as correctional officers or in institutional governance reported fewer hours of sleep on work nights and during off-shift nights than probation/parole officers on work night and off-shift nights, or than participants working in institutional wellness on work and off-shift nights. CWs reported feeling rested upon waking only 2.1 days (out of 7) per week on average. The mean quality rating of sleep was 1.6, indicating overall sleep quality in the poor to fair range. Correctional officers reported fewer days waking up per week feeling rested than institutional wellness employees. Correctional officers and probation/parole officers also reported a lower overall quality of sleep rating than participants working in institutional wellness. Many CWs from each occupational category reported symptoms consistent with clinical insomnia. Most participants (64.9%) screened positive on the ISI for insomnia. There were statistically significant differences in both positive screens for insomnia and on mean ISI scores across some occupational groups; specifically, correctional officers and participants working in institutional governance reported a higher prevalence of screening positive for insomnia than participants working in institutional wellness. Correctional officers also reported higher mean ISI scores than participants working in institutional wellness.

**Table 2:** Mean scores on sleep quality screening measures and frequency of insomnia positive screens by correctional worker occupational category.

Sleep quality measure	Total Sample	Wellness	Training	Governance	Correctional Officer	Probation/Parole Officer	Administration
Positive screens for insomnia, % (n)	64.9 (612)	52.6 (40)	60.6 (20)	72.2 (65)	66.9 (374)	60.3 (94)	65.5 (19)
Insomnia (ISI), mean (SD)	12.39 (6.58)	10.12^a^ (6.66)	12.45 (7.05)	12.60 (6.09)	13.04^b^ (6.64)	11.35 (6.41)	10.83 (4.89)
Hours of sleep work nights, mean (SD)	6.03 (1.21)	6.55^a^ (1.16)	6.17 (1.40)	5.85^b^ (1.28)	5.82^b^ (1.16)	6.58^a^ (1.10)	6.05 (1.08)
Hours of sleep off-shift night, mean (SD)	7.17 (1.74)	7.95^a^ (1.58)	7.55 (2.09)	6.66^b^ (1.91)	7.02^b^ (1.73)	7.55^a^ (1.56)	7.16 (1.37)
Days a week feeling rested, mean (SD)	2.09 (1.88)	2.71^a^ (1.80)	2.39 (2.16)	1.97 (2.07)	1.97^b^ (1.82)	2.23 (1.97)	1.86 (1.46)
Quality of sleep rating^1^, mean (SD)	1.64 (0.99)	2.14^a^ (0.95)	1.70 (1.13)	1.61 (0.96)	1.53^b^ (0.97)	1.78^b^ (1.04)	1.59 (0.78)

*ISI* Insomnia Severity Index. Differences in continuous measures across occupational groups were tested using ANOVA with Bonferroni post-hoc tests. Estimates with different superscripts differ from one another at *p* < 0.05 (i.e., indicate significant differences between occupational groups).

^1^Quality of sleep measured on a 5 point scale ranging from 0 (very poor) to 4 (very good).

Logistic regression analytic results are provided in Table 3 (dichotomous insomnia variable as independent variable) and Table 4 (total insomnia score as independent variable). Logistic regression analyses indicated that a positive screen for insomnia was associated with statistically significantly increased odds of screening positive for any mental health disorder in the total sample (Odds Ratio [OR] = 9.07, 95% Confidence Interval [CI] = 6.43, 12.80) and across most occupational categories (ORs ranged from 5.74 to 12.84; see Table 3) after adjustment for levels of work stress due to shift work.

**Table 3:** Logistic regression for association between positive screens for insomnia and positive screens for any current mental disorder by correctional worker occupational category.

Occupational category	β	SE	LR Chi square	OR	OR 95% CI		*p*-value
					Lower	Upper	
All correctional workers	2.35	0.17	226.75	10.49	7.50	14.67	< 0.001
Wellness	1.76	0.55	11.67	5.84	2.00	17.02	0.001
Training	–	–	–	–	–	–	–
Governance	2.73	0.69	21.31	15.33	3.94	59.62	< 0.001
Correctional officer	2.36	0.22	137.19	10.58	6.86	16.34	< 0.001
Probation/parole officer	2.10	0.40	30.65	8.14	3.70	17.90	< 0.001
Administration	–	–	–	–	–	–	–

**Table 4:** Logistic regression for association between total insomnia score and positive screens for any current mental disorder by correctional worker occupational category.

Occupational category	β	SE	LR Chi square	OR	OR 95% CI		*p*-value
					Lower	Upper	
All correctional workers	0.25	0.02	327.23	1.28	1.24	1.33	< 0.001
Wellness	0.20	0.05	21.78	1.23	1.11	1.36	< 0.001
Training	0.58	0.26	22.27	1.79	1.08	2.98	0.025
Governance	0.35	0.08	38.04	1.41	1.21	1.65	< 0.001
Correctional officer	0.25	0.02	203.66	1.28	1.23	1.34	< 0.001
Probation/parole officer	0.21	0.04	37.91	1.24	1.14	1.35	< 0.001
Administration	0.42	0.17	12.64	1.52	1.09	2.12	0.013

The relationship between a positive screening for insomnia and any positive screen for a mental health disorder could not be assessed among participants working in the institutional training or institutional administration categories due to low cell count sizes and insufficient outcome variability. A similar pattern of results was noted when examining the relationship between a positive screen for insomnia and individual mental health disorders (see supplementary online material (Table S1) for results across individual mental health disorders).

We also assessed for relationships between the total ISI score and screening positive for any mental health disorder (see Table 4). In the total sample, results indicated that each unit increase in the total insomnia scale score was associated with a statistically significant increase in the odds of screening positive for any mental health disorder (OR = 1.28, 95% CI = 1.24, 1.33) after adjustment for levels of work stress due to shift work. A similar pattern was noted across all occupational groups (ORs ranged from 1.23 to 1.73). A similar pattern of results when examining individual mental health disorders was noted; specifically, the relationship between total ISI score and individual mental health disorders (see supplementary online material (Table S2) for results across individual mental health disorders).

Correlational analytic results are provided in Tables 5 and 6. Total ISI scores were positively correlated with total scores for the PCL-5, the PHQ-9, the GAD-7, the PDSS, and the AUDI measures (see Table 5). The level of stress associated with shift work was inversely correlated with the additional quality of sleep indicators including total hours of sleep on work night, total hours of sleep on off-shift nights, days a week feeling rested, and overall quality of sleep rating.

**Table 5:** Correlations between insomnia and mental disorder screening measures across all correctional workers.

Mental disorder screening measure	ISI	PCL-5	PHQ-9	GAD-7	PDSS
PCL-5	0.554***	–	–	–	–
PHQ-9	0.684***	0.704***	–	–	–
GAD-7	0.620***	0.705***	0.803***	–	–
PDSS	0.477***	0.598***	0.649***	0.686***	–
AUDIT	0.178***	0.195***	0.175***	0.172***	0.121***

*ISI* Insomnia Severity Index, *PCL-5* Post-traumatic Stress Disorder Checklist for DSM-5, *PHQ-9* Patient Health Questionnaire, *GAD-7* Generalized Anxiety Disorder Scale, *PDSS-SR* Panic Disorder Symptoms Severity Scale, Self-Report, *AUDIT* Alcohol Use Disorders Identification Test.

*p < 0.05; **p < 0.01; ***p < 0.001.

**Table 6:** Correlations between sleep quality measures and stress regarding shift work.

Sleep quality measure	Shift work stress^1^	Hours of sleep work nights	Hours of sleep off-shift nights	Days a week feeling rested
Hours of sleep work nights	− 0.245***	–	–	–
Hours of sleep off-shift nights	− 0.073*	0.435***	–	–
Days a week feeling rested	− 0.250***	0.363***	0.157***	–
Quality of sleep rating^2^	− 0.306***	0.495***	0.233***	0.606***

^1^Shift work stress is measured on a 7-point Likert scale ranging from 0 (no stress) to 6 (a lot of stress).

^2^Quality of sleep measured on a 5-point Likert scale ranging from 1 (very poor) to 5 (very good).

*p < 0.05; **p < 0.01; ***p < 0.001.

## Discussion

The nature of correctional work impacts several aspects of CW well-being including job performance, physical health outcomes, mental health, and suicidal thoughts and behaviours^1,2,13^. The current study focused on the lesser-explored effects of correctional work on the duration and quality of sleep patterns and associated symptoms of various mental health disorders. More than half of the current CW sample screened positive for insomnia. There was evidence of a higher prevalence of insomnia (and poor sleep quality) among correctional officers when compared to CW employees working in institutional wellness. Most CWs reported obtaining only a poor or fair quality of sleep each night. Insomnia symptom scores were positively associated with screening positive for one or more mental health disorders, which is consistent with prior research^15^.

Most participants reported poor overall sleep quality and screened positive for insomnia. There were statistically significant differences across the occupational categories of CWs, suggesting that there may be distinct risk patterns of sleep disturbances associated with different roles. Most participants were institutional correctional officers who may have greater variability in work hours, schedules, and increased requirements to work shifts or overtime during staff shortages. The current results appear consistent with other studies that suggest sleep patterns among all Canadian PSP may be impacted by work hours, schedules, and increased requirements to work shifts^6,38^. In the current study, the odds of screening positive for any mental health disorder were associated with disrupted sleep quantity and quality, and the prevalence of positive screens for insomnia were similar to results from studies with diverse PSP^15^, police officers^39,40^, and firefighters^41^. Future research should explore potential associations between different types or patterns of shift schedules, the level of reported insomnia and poor sleep quality, and interactions with sex and gender. We further recommend qualitative explorations of CW sleep patterns and quality to better understand the social and organizational contexts underpinning CW experiences with sleep and mental health.

The National Sleep Foundation guidelines recommended that adults work to achieve 7–9 h of sleep on a nightly basis for optimal physical and mental well-being^42^. There is a bidirectional relationship between sleep and mental health documented in non-correctional populations wherein nighttime sleep disturbances interfere with daytime mood and cognitive processes, which then contribute to difficulties falling or staying asleep^22^. Insomnia also appears to be a transdiagnostic^22^ and precursory factor for several mental health disorders^15,22,43^. Interventions that target insomnia may also help to reduce the diverse mental health challenges among PSP, particularly CWs. Policies and organizational practices that explicitly target sleep hygiene behaviours must consider shift length, pattern, and work hours, along with sleeping timing and exercise^44–47^ as part of organizational efforts to improve CW sleep quality and therein mental health and well-being. Implementing evidence-based organizational policies regarding shift work and integrating educational awareness campaigns about proper sleep hygiene practices into PSP training programs^15^ may serve as protective factors against the development of negative health outcomes. Support from supervisors and increased job autonomy are also important factors in making CWs feel safe and secure within the confines of their often stressful workplaces^2^. General mental health improvement and behavioural interventions have demonstrated effectiveness for improving sleep quality and quantity, with most treatment programs for insomnia focusing on a range of cognitive, emotional, and behavioural interventions^48^.

### Limitations

The current study has several limitations that help inform directions for future research. The sample was self-selected and the response rate cannot be definitively calculated; as such, the results may not generalize to provincial and territorial CW populations in other Canadian regions. The current study was Ontario specific, which may limit generalizability to other jurisdictions, including the Canadian federal correctional service (Correctional Service Canada), which is the government agency responsible for servicing justice-involved populations serving court-imposed sentences of a term of two years or more. The sample used in the current study was not a random probability sample of provincial CWs in the province of Ontario, which limits our ability to make inferences even in this population. Responses were based on anonymous online self-reported data; as such, despite findings being consistent with previous related research^15^, clinical reliability and validity remain ambiguous. Participants may also under- or over-report clinical symptoms because of mental health stigma or confidentiality concerns that still permeate some correctional workplaces^49^.

Future research would benefit from replication with structured clinical interviews. Sleep problems (i.e., having trouble falling/staying asleep) are also included as mental health disorder symptoms on the mental health disorder screens used in the current study (i.e., on the PTSD and MDD screens), which could have over-estimated relationships between sleep problems and mental health disorders. The insomnia, sleep quality, and mental health disorder measures used in the study also largely represented current functioning (time frames assessed ranged from past 12 months to the past 7 days), and we also did not have information on the onset of insomnia, sleep problems, or mental health disorders. Therefore, we cannot determine whether the onset of these issues occurred before or after respondents entered correctional work. This work is also based on the assumption that insomnia predicts mental health disorders, and it could be that mental health disorders also contribute to insomnia or other sleep problems, or that bidirectional relationships exist. Lastly, the data were cross-sectional, precluding discussions of causality; thus, future CW sleep research would benefit from using prospective longitudinal designs.

## Conclusion

The current study highlights the relationship between sleep quality and mental health disturbances in a population that necessarily navigates pervasive psychological and physical stressors. The current study replicates and extends previous research regarding sleep and mental health among PSP. The results underscore that CWs experience substantial challenges with sleep that were associated with symptoms of several mental health disorders. The results also showcased differences between CW occupational categories. Correctional officers reported the most difficulties with insomnia, but more than half of participants in each category screened positive for insomnia. CWs reported waking up feeling rested on less than 3 days in a normal week and the stress of shift work was inversely correlated with sleep quality. Institutional correctional officers and institutional governance workers reported less sleep on work nights than CWs working in wellness, training, probation and parole, and administration. Overall, the results call for tailored interventions and policy changes that may help to improve sleep quality and reduce the mental health challenges experienced by CWs.

### Strengths and limitations

The current study is the first to examine sleep disorders among correctional workers across diverse occupational categories.

- The current study is comprehensive and includes a vast sample of a mixed group of correctional workers.
- The study is limited in that we cannot report on a sampling frame.
- The study is limited as the data are self-reported rather than based on diagnostic interviews.

### Supplementary Information

Supplementary Tables.

## Data Availability

The datasets generated and/or analysed during the current study are not publicly available due to ethics and research agreements with diverse services but are available from the corresponding author on reasonable request.
