# Supplementary material for: Sleep quality and mental disorder symptoms among correctional workers in Ontario, Canada
Source: Sci Rep. 2024 Jun 28;14:14963. doi: 10.1038/s41598-024-65891-8 (PMC11213858; doi:10.1038/s41598-024-65891-8)
Supplement: Supplementary file 1 — Supplementary Tables. [file 41598_2024_65891_MOESM1_ESM.docx]

| **Table S1.** Logistic Regression for Association between Positive Screens for Insomnia and Positive Screens for Any Current Mental Health Disorder and Individual Mental Health Disorders by Correctional Worker Occupational Category | | | | | | | |
| --- | --- | --- | --- | --- | --- | --- | --- |
| **Occupational Category** | **β** | **SE** | **LR Chi Square** | **AOR** | **AOR 95% CI** | | ***p*-value** |
|  |  |  |  |  | **Lower** | **Upper** |  |
| Any Mental Disorder |  |  |  |  |  |  |  |
| All Correctional Workers | 2.35 | 0.17 | 226.75 | 10.49 | 7.50 | 14.67 | < .001 |
| Wellness | 1.76 | 0.55 | 11.67 | 5.84 | 2.00 | 17.02 | .001 |
| Training | --- | --- | --- | --- | --- | --- | --- |
| Governance | 2.73 | 0.69 | 21.31 | 15.33 | 3.94 | 59.62 | < .001 |
| Correctional Officer | 2.36 | 0.22 | 137.19 | 10.58 | 6.86 | 16.34 | < .001 |
| Probation/Parole Officer | 2.10 | 0.40 | 30.65 | 8.14 | 3.70 | 17.90 | < .001 |
| Administration | --- | --- | --- | --- | --- | --- | --- |
| Post-Traumatic Stress Disorder |  |  |  |  |  |  |  |
| All Correctional Workers | 1.55 | 0.21 | 111.36 | 4.71 | 3.14 | 7.07 | < .001 |
| Wellness | 1.08 | 0.74 | 7.58 | 2.93 | 0.69 | 12.47 | .146 |
| Training | --- | --- | --- | --- | --- | --- | --- |
| Governance | 2.68 | 1.07 | 14.74 | 14.59 | 1.78 | 119.78 | .013 |
| Correctional Officer | 1.39 | 0.25 | 61.12 | 4.02 | 2.45 | 6.60 | < .001 |
| Probation/Parole Officer | 1.45 | 0.53 | 13.96 | 4.26 | 1.51 | 11.99 | .006 |
| Administration | --- | --- | --- | --- | --- | --- | --- |
| Major Depressive Disorder |  |  |  |  |  |  |  |
| All Correctional Workers | 2.59 | 0.23 | 229.25 | 13.39 | 8.46 | 21.19 | < .001 |
| Wellness | --- | --- | --- | --- | --- | --- | --- |
| Training | --- | --- | --- | --- | --- | --- | --- |
| Governance | 3.12 | 1.07 | 20.80 | 22.54 | 2.76 | 184.03 | .004 |
| Correctional Officer | 2.47 | 0.30 | 138.07 | 11.86 | 6.54 | 21.50 | < .001 |
| Probation/Parole Officer | 1.92 | 0.43 | 28.63 | 6.85 | 2.91 | 16.12 | < .001 |
| Administration | --- | --- | --- | --- | --- | --- | --- |
| Generalized Anxiety Disorder |  |  |  |  |  |  |  |
| All Correctional Workers | 2.30 | 0.24 | 178.07 | 10.02 | 6.22 | 16.15 | < .001 |
| Wellness | 2.10 | 0.69 | 13.84 | 8.19 | 2.12 | 31.67 | .002 |
| Training | --- | --- | --- | --- | --- | --- | --- |
| Governance | --- | --- | --- | --- | --- | --- | --- |
| Correctional Officer | 2.47 | 0.36 | 135.54 | 11.79 | 5.78 | 24.08 | < .001 |
| Probation/Parole Officer | 1.49 | 0.42 | 19.58 | 4.43 | 1.93 | 10.12 | < .001 |
| Administration | --- | --- | --- | --- | --- | --- | --- |
| Panic Disorder |  |  |  |  |  |  |  |
| All Correctional Workers | 2.29 | 0.40 | 74.96 | 9.85 | 4.49 | 21.64 | < .001 |
| Wellness | 2.01 | 0.82 | 9.23 | 7.47 | 1.49 | 37.33 | .014 |
| Training | --- | --- | --- | --- | --- | --- | --- |
| Governance | --- | --- | --- | --- | --- | --- | --- |
| Correctional Officer | 2.46 | 0.60 | 51.10 | 11.73 | 3.58 | 38.37 | < .001 |
| Probation/Parole Officer | 1.52 | 0.80 | 17.00 | 4.57 | 0.96 | 21.90 | .057 |
| Administration | --- | --- | --- | --- | --- | --- | --- |
| Alcohol Use Disorder |  |  |  |  |  |  |  |
| All Correctional Workers | 1.26 | 0.42 | 26.25 | 3.54 | 1.56 | 8.04 | .003 |
| Wellness | 2.01 | 0.82 | 9.23 | 7.47 | 1.49 | 37.33 | .014 |
| Training | --- | --- | --- | --- | --- | --- | --- |
| Governance | 0.65 | 1.19 | 0.50 | 1.92 | 0.19 | 19.66 | .581 |
| Correctional Office | 1.05 | 0.47 | 14.27 | 2.85 | 1.15 | 7.10 | .024 |
| Probation/Parole Officer | --- | --- | --- | --- | --- | --- | --- |
| Administration | --- | --- | --- | --- | --- | --- | --- |

*Notes.* AOR = adjusted odds ratio (i.e., odds ratio adjusted for level of work stress due to shift work). Dashes in cells indicate analyses that could not be run due to insufficient cell count sizes and insufficient outcome variability.

| **Table S2.** Logistic Regression for Association between Total Insomnia Score and Positive Screens for Any Current Mental Health Disorder and Individual Mental Health Disorders by Correctional Worker Occupational Category | | | | | | | |
| --- | --- | --- | --- | --- | --- | --- | --- |
| **Occupational Category** | **β** | **SE** | **LR Chi Square** | **AOR** | **AOR 95% CI** | | ***p*-value** |
|  |  |  |  |  | **Lower** | **Upper** |  |
| Any Mental Disorder |  |  |  |  |  |  |  |
| All Correctional Workers | 0.25 | 0.02 | 327.23 | 1.28 | 1.24 | 1.33 | < .001 |
| Wellness | 0.20 | 0.05 | 21.78 | 1.23 | 1.11 | 1.36 | < .001 |
| Training | 0.58 | 0.26 | 22.27 | 1.79 | 1.08 | 2.98 | .025 |
| Governance | 0.35 | 0.08 | 38.04 | 1.41 | 1.21 | 1.65 | < .001 |
| Correctional Officer | 0.25 | 0.02 | 203.66 | 1.28 | 1.23 | 1.34 | < .001 |
| Probation/Parole Officer | 0.21 | 0.04 | 37.91 | 1.24 | 1.14 | 1.35 | < .001 |
| Administration | 0.42 | 0.17 | 12.64 | 1.52 | 1.09 | 2.12 | .013 |
| Post-Traumatic Stress Disorder |  |  |  |  |  |  |  |
| All Correctional Workers | 0.15 | 0.01 | 166.70 | 1.16 | 1.13 | 1.20 | < .001 |
| Wellness | 0.15 | 0.06 | 12.53 | 1.16 | 1.03 | 1.30 | .012 |
| Training | 0.17 | 0.11 | 4.69 | 1.18 | 0.95 | 1.46 | .129 |
| Governance | 0.15 | 0.05 | 13.60 | 1.16 | 1.05 | 1.29 | .004 |
| Correctional Officer | 0.14 | 0.02 | 97.63 | 1.15 | 1.11 | 1.20 | < .001 |
| Probation/Parole Officer | 0.19 | 0.04 | 29.87 | 1.21 | 1.11 | 1.32 | < .001 |
| Administration^1^ | 0.42 | 0.17 | 12.64 | 1.52 | 1.09 | 2.12 | .013 |
| Major Depressive Disorder |  |  |  |  |  |  |  |
| All Correctional Workers | 0.26 | 0.02 | 356.25 | 1.30 | 1.25 | 1.35 | < .001 |
| Wellness | 0.30 | 0.08 | 30.28 | 1.34 | 1.15 | 1.58 | < .001 |
| Training | 0.32 | 0.11 | 16.18 | 1.37 | 1.10 | 1.73 | .006 |
| Governance | 0.28 | 0.07 | 32.71 | 1.33 | 1.16 | 1.52 | < .001 |
| Correctional Officer | 0.26 | 0.02 | 219.35 | 1.29 | 1.23 | 1.36 | < .001 |
| Probation/Parole Officer | 0.23 | 0.04 | 49.01 | 1.26 | 1.16 | 1.38 | < .001 |
| Administration^1^ | 0.46 | 0.18 | 14.55 | 1.59 | 1.11 | 2.26 | .011 |
| Generalized Anxiety Disorder |  |  |  |  |  |  |  |
| All Correctional Workers | 0.22 | 0.02 | 273.89 | 1.24 | 1.20 | 1.29 | < .001 |
| Wellness | 0.21 | 0.06 | 20.97 | 1.24 | 1.10 | 1.39 | < .001 |
| Training | 0.18 | 0.09 | 7.63 | 1.20 | 1.01 | 1.43 | .038 |
| Governance | 0.36 | 0.08 | 36.82 | 1.43 | 1.21 | 1.69 | < .001 |
| Correctional Officer | 0.21 | 0.02 | 188.66 | 1.24 | 1.19 | 1.30 | < .001 |
| Probation/Parole Officer | 0.18 | 0.04 | 35.16 | 1.20 | 1.11 | 1.29 | < .001 |
| Administration^1^ | 0.32 | 0.16 | 6.90 | 1.38 | 1.01 | 1.89 | .041 |
| Panic Disorder |  |  |  |  |  |  |  |
| All Correctional Workers | 0.18 | 0.02 | 124.26 | 1.20 | 1.15 | 1.25 | < .001 |
| Wellness | 0.16 | 0.06 | 11.46 | 1.18 | 1.05 | 1.32 | .004 |
| Training^1^ | 0.19 | 0.12 | 3.87 | 1.22 | 0.96 | 1.55 | .101 |
| Governance | 0.16 | 0.07 | 7.47 | 1.17 | 1.02 | 1.35 | .026 |
| Correctional Officer | 0.19 | 0.03 | 82.81 | 1.21 | 1.15 | 1.27 | < .001 |
| Probation/Parole Officer | 0.23 | 0.06 | 31.65 | 1.26 | 1.12 | 1.41 | < .001 |
| Administration^1^ | 0.17 | 0.16 | 1.39 | 1.19 | 0.86 | 1.63 | .290 |
| Alcohol Use Disorder |  |  |  |  |  |  |  |
| All Correctional Workers | 0.06 | 0.02 | 21.73 | 1.06 | 1.02 | 1.11 | .007 |
| Wellness | 0.16 | 0.06 | 11.46 | 1.18 | 1.05 | 1.32 | .004 |
| Training^1^ | 0.22 | 0.20 | 1.65 | 1.24 | 0.84 | 1.84 | .282 |
| Governance | 0.06 | 0.07 | 0.74 | 1.06 | 0.91 | 1.23 | .451 |
| Correctional Office | 0.04 | 0.02 | 10.64 | 1.04 | 0.99 | 1.10 | .115 |
| Probation/Parole Officer | 0.13 | 0.06 | 4.43 | 1.14 | 1.01 | 1.28 | .038 |
| Administration | --- | --- | --- | --- | --- | --- | --- |

*Notes.* AOR = adjusted odds ratio (i.e., odds ratio adjusted for level of work stress due to shift work). Dashes in cells indicate analyses that could not be run due to insufficient cell count sizes and insufficient outcome variability. Dashes in cells indicate analyses that could not be run due to insufficient cell count sizes and insufficient outcome variability.

^1^Models were run unadjusted due to a lack of variability in levels of work stress due to shift work and the outcome variable.
